# Editorial: The role of astrocytes in stroke

**DOI:** 10.3389/fncel.2023.1205798

**Published:** 2023-05-17

**Authors:** Wen-Jun Tu, Anwen Shao, Yi Huang

**Affiliations:** ^1^Department of Neurosurgery, Beijing Tiantan Hospital, Capital Medical University, Beijing, China; ^2^Geriatrics Innovation Center, Weifang People's Hospital, Weifang, China; ^3^Institute of Radiation Medicine, Chinese Academy of Medical Sciences and Peking Union Medical College, Tianjin, China; ^4^Department of Neurosurgery, Second Affiliated Hospital, School of Medicine, Zhejiang University, Zhejiang, China; ^5^Department of Neurosurgery, The First Affiliated Hospital of Ningbo University, Ningbo, China

**Keywords:** stroke, astrocytes, ischemic stroke, subarachnoid hemorrhage (SAH), intracerebral hemorrhage

Stroke is a major health concern worldwide and continues to be the second-leading cause of death and the third-leading cause of death and disability combined (Feigin et al., 2022). The estimated global cost of stroke is over US$721 billion, which accounts for 0.66% of the global Gross Domestic Product (GDP; Feigin et al., 2022). In China, stroke is associated with the highest loss of disability-adjusted life-years of any disease (Wu et al., 2019) and the prevalence continues to rise in the last decade (Tu et al., 2022a). Shockingly, in 2020 alone, there were 17.8 million cases of stroke, 3.4 million new strokes, and 2.3 million stroke-related deaths (Tu et al., 2023). Despite the Chinese government's continued investment in stroke prevention, treatment, and rehabilitation (Wu et al., 2019; Zhao et al., 2019; Chao et al., 2021), the burden of stroke in China remains significant and is one of the major risk factors for the health of the population, particularly as the aging process continues to progress (Tu et al., 2022b).

Astrocytes are crucially involved in the most integrated functions of the central nervous system, which are not only necessary for the normal functioning of the brain but are also critically involved in many pathological conditions, including stroke (Anderson et al., 2003). Following a stroke, astrocytes perform multiple functions, some of which can be detrimental, while others can be beneficial to neurological recovery (Liu and Chopp, 2016). Astrocytes play a key role in mediating brain network connectivity, plasticity, and neurological recovery after a stroke (Li et al., 2014). In the acute stages of ischemic stroke, astrocytes are critical for the reconstruction of the blood-brain barrier and for neuroprotection, as they increase the uptake of extracellular glutamate and sodium/potassium-ATPase activity (Patabendige et al., 2021). Studies have shown that astrocytes not only influence cell survival but also contribute to angiogenesis, neuronal plasticity, and functional recovery in the days to weeks after stroke (Zhao and Rempe, 2010). Reactive astrocytes exhibit high plasticity, and modulation of local reactive astrocytes has shown promise as a strategy for cell-based therapy for stroke (Choudhury and Ding, 2016). Research by Williamson et al. (2021) has demonstrated that reactive astrocytes are crucial for vascular repair and remodeling after ischemic stroke in mice. Jiang et al. (2021) found that converting reactive astrocytes to induced neurons can enhance neuronal repair and functional recovery after ischemic stroke in mice. Reactive astrocytes provide neuroprotection in the acute phase of ischemic stroke through antioxidation, antiexcitatory effects, and metabolic support (He et al., 2022). At the same time, reactive astrocytes also play a vital role in neuroinflammation and brain edema by communicating with microglia and endothelial cells (He et al., 2022).

Ischemic stroke activates astrocytes, which play a crucial double-edged sword role (Han et al., 2022). On one hand, astrocytes play a protective role by releasing anti-inflammatory, antioxidant, and neurotrophic factors. On the other hand, they release neurotoxic substances that damage the blood-brain barrier and increase the area of cerebral infarction. Although various drugs have shown positive effects on recovery after stroke by targeting astrocytes, most of these drugs are still in the experimental stage (Han et al., 2022). Further understanding of the role of astrocytes in the pathogenesis of stroke may provide new insights into developing novel therapeutic approaches, which can be tested alongside neuronal rescue and repair strategies.

This Research Topic attracted original research and review articles related to “*The role of astrocytes in stroke*”, covering molecular and cellular mechanisms to clinical translational applications. Six articles were published, including three original research studies and three reviews. One original research study investigated the impact of glymphatic drainage blocking on brain edema and neuroinflammation in an experimental intracerebral hemorrhage model in rats (Liu et al.). Another study found that 14,15-Epoxyeicosatrienoic Acid could protect human cerebral microvascular endothelial cells against oxygen glucose deprivation/re-oxygenation (OGD/R) damage (Qu et al.), while the third study confirmed the neuroprotective effects of 2-Methoxyestradiol in rats with early brain injury after subarachnoid hemorrhage (Hu et al.). The three review articles covered intracerebral hemorrhage (ICH)-induced cell death, reactive astrocytes' specific role in stroke, and the roles of microRNAs in astrocytes after cerebral stroke (Li et al.; Zhang, Khan et al.; Zhang, Lei et al.). These studies enriched our understanding of the relationship between astrocytes and stroke and provided valuable insights into effective treatment options for stroke. We extend our deepest gratitude to all the contributing authors, reviewers, and editors who participated in this Research Topic.

## Author contributions

W-JT: analysis and interpretation of data, drafting of the manuscript, and statistical analysis. All authors: study concept and design, acquisition of data, critical revision of the manuscript for important intellectual content, and administrative, technical, or material support. All authors contributed to the article and approved the submitted version.
